# Oxygen Sensing Neurons and Neuropeptides Regulate Survival after Anoxia in Developing *C. elegans*

**DOI:** 10.1371/journal.pone.0101102

**Published:** 2014-06-26

**Authors:** John J. Flibotte, Angela M. Jablonski, Robert G. Kalb

**Affiliations:** 1 Department of Pediatrics, Division of Neonatology, Children’s Hospital of Philadelphia, Philadelphia, Pennsylvania, United States of America; 2 Department of Neuroscience, Perelman School of Medicine, University of Pennsylvania, Philadelphia, Pennsylvania, United States of America; 3 Department of Pediatrics, Division of Neurology, Research Institute, Children’s Hospital of Philadelphia, Philadelphia, Pennsylvania, United States of America; 4 Department of Neurology, Perelman School of Medicine, University of Pennsylvania, Philadelphia, Pennsylvania, United States of America; Brown University/Harvard, United States of America

## Abstract

Hypoxic brain injury remains a major source of neurodevelopmental impairment for both term and preterm infants. The perinatal period is a time of rapid transition in oxygen environments and developmental resetting of oxygen sensing. The relationship between neural oxygen sensing ability and hypoxic injury has not been studied. The oxygen sensing circuitry in the model organism *C. elegans* is well understood. We leveraged this information to investigate the effects of impairments in oxygen sensing on survival after anoxia. There was a significant survival advantage in developing worms specifically unable to sense oxygen shifts below their preferred physiologic range via genetic ablation of BAG neurons, which appear important for conferring sensitivity to anoxia. Oxygen sensing that is mediated through guanylate cyclases (*gcy-31, 33, 35*) is unlikely to be involved in conferring this sensitivity. Additionally, animals unable to process or elaborate neuropeptides displayed a survival advantage after anoxia. Based on these data, we hypothesized that elaboration of neuropeptides by BAG neurons sensitized animals to anoxia, but further experiments indicate that this is unlikely to be true. Instead, it seems that neuropeptides and signaling from oxygen sensing neurons operate through independent mechanisms, each conferring sensitivity to anoxia in wild type animals.

## Introduction

Hypoxic brain injury remains a major cause of future neurodevelopmental impairment in newborns. Incidence of hypoxic ischemic encephalopathy among terms infants is estimated to be 3–5/1000 births, and is even higher in preterm populations [1], [2]. Despite the magnitude of this problem, the only treatment known to be effective is therapeutic hypothermia. However, the benefit conferred is incomplete, offering only a 30% improvement in neurodevelopmental outcome and is largely restricted to infants with moderate injury [3]. Furthermore, this treatment is limited to those infants born 35 weeks and older, leaving no specific treatment for preterm infants [4]. There is an urgent need for greater understanding of the molecular and cellular events that contribute to hypoxic brain injury in order to develop novel neuroprotective therapies for these infants.

Much experimental work indicates that the susceptibility of a cell to hypoxic insult is controlled by its inherent properties such as: 1) the balance between the cell’s metabolic rate and oxygen availability, 2) transcriptional response to oxygen deprivation, or 3) density of membrane channels conferring sensitivity to cytotoxicity [1], [3], [5]. Such factors are certainly important determinants; however, the possibility of non cell-autonomous signaling driving the response and ability to resist hypoxic injury is increasingly being explored [3], [6]. These signals are particularly attractive as targets of novel therapeutics.

We used the model organism *Caenorhabditis elegans* (*C. elegans*) to ask whether sensory neurons that respond to changes in oxygen levels modify systemic response to anoxic injury in a non cell-autonomous fashion. *C. elegans* is a free living soil nematode that prefers environments with oxygen concentrations ranging between 5 and 12% [4], [7]. Specific oxygen sensing neurons are activated and drive the animal’s response to a preferred envionrment. URX, AQR and PQR neurons respond to oxygen shifts above the physiologic range and BAG neurons respond to oxygen shifts below the physiologic range [7]–[9]. Specific guanylate cyclases (GCs) within these neurons transduce the changes in ambient oxygen to biologic response [7], [9], [10]. We evaluated survival after anoxia in worms mutated in various aspects of this sensory system. In addition, based on other investigators’ findings that neuropeptides are able to modify cellular response to environmental stress [11], we evaluated whether the ability of neurons to signal using neuropeptides influenced survival. Our findings suggest that the survival of developing animals to an anoxic insult is determined in part by signals originating in oxygen sensing neurons, as well as by neuropeptide signals.

## Methods

### Worm Strains Used

Worm strains used are listed in **Table 1** **.** Worm strains with mutations in guanylate cyclases were genotyped by polymerase chain reaction (PCR) and sequencing to confirm identity.

**Table 1:** A list of the strains of worms used in this study, the genotype for each strain, and a brief description of functional impairments.

Strain	Genotype	Description/FunctionalImpairment	#N2Controls	#AnimalsEvaluated	#Trials	Figure
CX11697	*kyIs536* *[Pflp-17::caspase* *p17::sl2::gfp;* *Pelt-* *2::nls::gfp];* *kyIs538* *[Pglb-5::caspase* *p12::sl2::gfp;* *Pelt-* *2::mCherry]*	Loss of BAG function	252	235	4	3a
CX7102	*lin-15(n765) qaIs2241* *[gcy-36::egl-1, gcy-* *35::gfp, lin-15(+)]*	Loss of URX/AQR/PQR function	496	522	8	3a
RK21	*gcy33 (ok232);* *oyIs18 [gcy-* *8*:: GFP*]*	Null allele in guanylatecyclase (GC) commonto URX/AQR/PQR andBAG neurons	784	718	12	4a
RK32	*gcy-35 (ok769)*;*oyIs18 [gcy-* *8*:: GFP*]*	Null allele in GCspecific forURX/AQR/PQRneurons	482	522	8	4a
RK33	*gcy-31 (ok296);* *oyIs18 [gcy-* *8*:: GFP*]*	Null allele in GCspecific for BAG neuron	295	328	5	4a
CZ3805	*gcy-33 (ok232); gcy-* *31 (ok296)*	Compound mutant GC alleles	261	271	4	4b
CX6803	*gcy-35 (ok769); gcy-33 (ok232); gcy-31 (ok296)*	Compound mutant GC alleles	337	323	5	4b
VC461	*egl-3 (gk238)*	Null allele forneuropeptide processingenzyme	548	560	9	5a
VC671	*egl-3 (ok979)*	Null allele forneuropeptide processingenzyme	392	439	7	5b
RK35	*unc31(e928); oyIs18 [gcy-* *8::GFP]*	Dense core vesiclefusion mutant	288	319	6	5c
MT150	*egl-3(n150ts)*	Temperature sensitiveEGL-3 null allele	225	242	5	5d
CX13480	*egl-3(n150ts);* *kyEx4050* *[flp-17::egl-* *3::sl2::gfp]*	Temperature sensitiveEGL-3 null allele withEGL-3 expressed inBAG neuron	189	262	5	5d
e1370	*daf-2(e1370)*	DAF-2 null allele	391	367	8	6
GR1307	*daf-* *16(mgDf50)*	DAF-16 null allele	119	156	3	6
GR1309	*daf-2(e1370); daf-* *16(mgDf50)*	DAF-2/DAF-16 double	261	233	5	6
RK31	*oyIs18 [gcy-* *8::GFP]*	GFP expressed inAFD neurons.	308	281	6	Supplement

The column indicating #N2 controls indicates the number of N2 animals that served as matched controls for each survival experiment. The #Animals Evaluated indicates the number of animals for each strain that were subjected to anoxia for survival determination. The #Trials indicates the number of independent experiments that formed the basis of survival estimates for each strain. The final column of the table indicates the figure where the survival data appear.

### Anoxic Exposure & Survival Assay

All worms were maintained on standard nematode growth medium (NGM) seeded with *Escherichia coli* (OP50) at 20 degrees Celsius, as has been previously described [12]. A synchronous population of worms was generated by either timed egg lay or bleaching. We allowed worms to develop to the L4 stage (larval stage just prior to adulthood), which was typically 48 hours after egg lay. In order to ensure developmental synchrony between strains, worms with a visible white vulva, characteristic of mid-L4, were individually picked onto fresh plates and subjected to anoxia.

**Figure 1:**
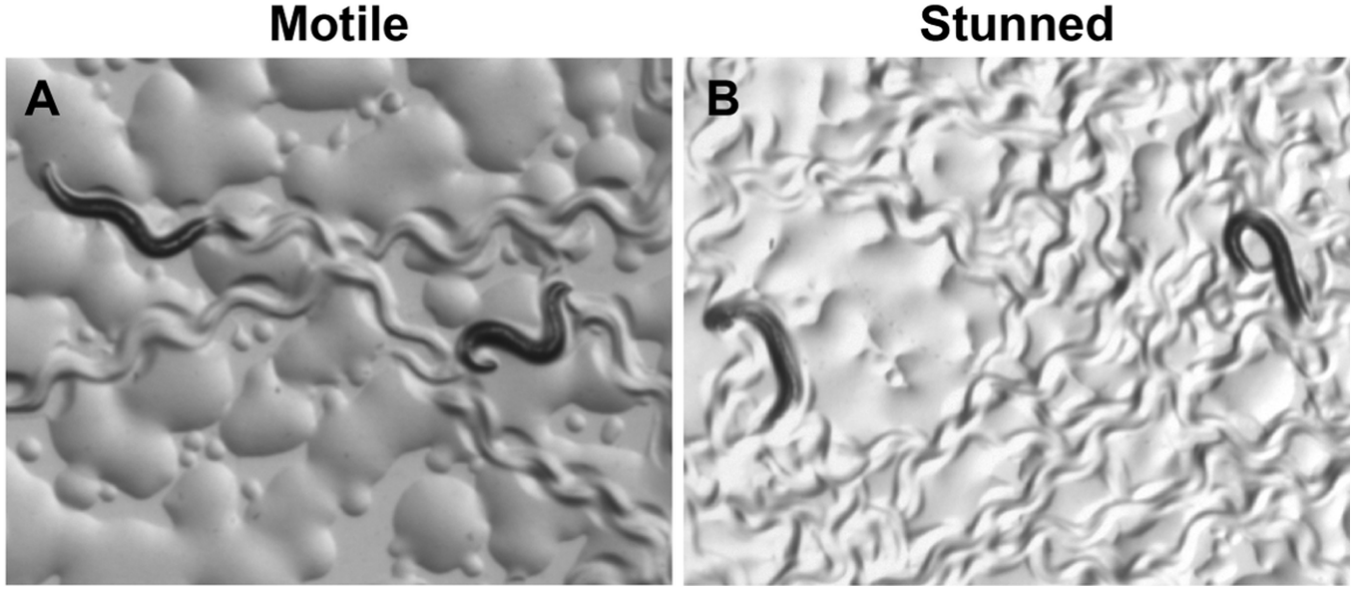
Worms Appear Stunned after Anoxia. Photographs of worms before (A) and after (B) anoxia. Prior to insult, animals are freely moving and their appearance has a characteristic sinusoidal shape as their bodies bend with locomotion. After anoxia, they enter suspended animation (“stunned”) and do not move. They appear either arched or as rods. This is a reversible state, and surviving animals will begin moving within 24 hours after being removed from the anoxia chamber.

For anoxic exposure, 40–80 L4 worms with the genotype of interest were picked onto a fresh NGM plate with OP50. In parallel, a second plate was generated of L4 wild type (N2) worms and the 2 plates were sealed into a Bio-Bag™ (Type A anaerobic environmental system, Becton-Dickinson). Anaerobic conditions (oxygen<0.001 kPa) were induced, as has been previously described [13], and were achieved within one hour of sealing the bag. This was confirmed by color change of a resazurin indicator (per manufacturer information). When we removed the plates from the bag, we observed that the indicators remained reversible. Duration of exposure is indicated for each experiment and occurred at 20 degrees Celsius. The one exception is the experiment conducted with *egl-3(n150ts)*, which is a temperature sensitive allele. For this experiment, anoxia was performed at 25 degrees Celsius for 24 hours. Data not shown indicated that this timepoint would produce 50% survival in the N2 animals. After exposure to anoxia, worms enter into a state of suspended animation (“stunned”) that is reversible and protective [14]. This is also easily observable on removal from the bag (**Figure 1**). We visually confirmed that all worms appeared “stunned” as an additional confirmation of adequate exposure to anoxia. Worms were allowed to recover for 24 hours before quantification of survival. Death was defined as absence of spontaneous or touch-evoked movement and a single investigator examined and quantified death in all strains in a blinded manner. Full details of all experiments are included in **Table 2**, including number of independent trials, number of controls compared, and number animals for each strain tested.

**Table 2:** Adapted from [9].

Neuron	Sensing Specificity	Specific Guanylate Cyclases
URX	Oxygen upshift	*gcy-33, gcy-35, gcy-36*
AQR	Oxygen upshift	*gcy-33, gcy-35, gcy-36*
PQR	Oxygen upshift	*gcy-33, gcy-35, gcy-36*
BAG	Oxygen downshift	*gcy-33, gcy-31*

Many of the strains used in our experiments also contained the integraged chromosomal array *oyIs18*, which labels AFD neurons with green fluorescent protein (GFP). These were used for experiments which are not reported in this paper. In order to ensure that the presence of this array itself did not influence our survival results, we specifically tested survival of worm strains containing *oyIs18* versus N2 animals in our anoxia experiments. There was no significant difference (*oyIs18* vs N2, 42.9% vs 50.7%, *p* = 0.35) (**Figure S1**).

### Heat Shock Survival Assay

Strains were synchronized via a timed egg lay. At L4 stage, ∼50 worms were synchronized by the transparency of the vulva. At one day post L4, worms were placed at 35 degrees Celsius. Every hour, all plates were removed and survival scored by the lack of response to touch and then all plates were returned to 35 degrees Celsius. Assay ended when all worms were dead. Assay was repeated 3 independent times using independent synchronizations on different days.

### Statistical Analysis

All data were analyzed in GraphPad Prism™ (version 6.0C for Mac OS X, GraphPad Software, La Jolla California USA, www.graphpad.com). We analyzed the pooled survival results using a paired two-tailed t-test to control for inter-bag variability in wild type survival. We report standard deviation rather than standard error of the mean because each experiment included only one technical replicate of each independent biologic strain. This was repeated several times per strain on different days (for full count see **Table 1**), and the reported mean is therefore an average survival of all independent replicates for each strain of interest. For heatshock survival assays, we analyzed mean survival using a one-way ANOVA. The threshold for significance was always set to *p*<0.05.

## Results

### Survival of the Wild Type (N2)

**Figure 2:**
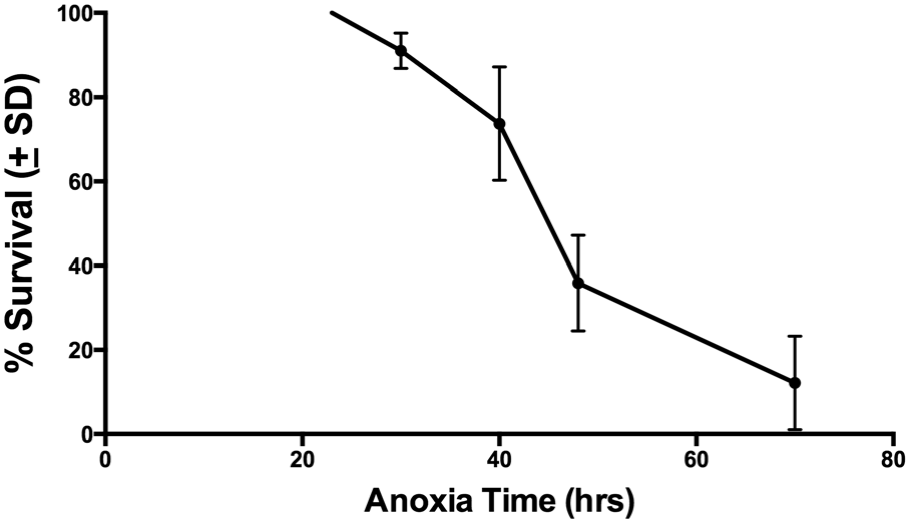
Wild Type (N2) Survival After Anoxia of Varying Duration. This figure shows the survival of wild type (N2) worms after varied periods of anoxia. Survival was evaluated after the following anoxia times: 24 h (2 trials, n = 202), 30 h (2 trials, n = 192), 40 h (11 trials, n = 775), 48 h (9 trials, n = 651) and 70 hours (2 trials, n = 162). Error bars represent standard deviation.

We are particularly interested in the responses of developing organisms to anoxic insult. Thus, in this study, all anoxic insults were imposed on larval stage 4 (L4) animals, which are immature, pre-reproductive organisms. We began by determining the survival of N2 (wild type) worms exposed to varying durations of anoxia. Our goal was to identify the length of time that would be lethal for approximately 50% of the N2 population. Results from these experiments appear in **Figure 2**. There is a steep drop in survival from 40 to 48 hours of anoxia, under our experimental conditions. Based on these data, we chose to expose L4 worms to 40–48 hours anoxia for the remaining experiments. In every experiment, we included N2 as controls within the same bag in order to directly compare survival of genotypes being tested.

### Impaired Neural Oxygen Sensing Improves Survival after Anoxia

We next evaluated the survival of worm strains with impairments in their oxygen sensing abilities. Oxygen sensing in *C. elegans* is coordinated by specialized sensory neurons that respond when environmental oxygen is shifted to a level higher or lower than the preferred physiologic range [7], [9]. The neurons URX, AQR and PQR respond to oxygen upshifts, while the BAG neuron responds to oxygen downshifts [7], [9].

We used two strains to evaluate the influence of impairments in oxygen sensing ability on survival after anoxia. *kyIs536* is a strain of worms with genetic ablation of the BAG neuron and therefore cannot sense oxygen downshifts. *qaIs2241* is a strain of worms with URX/AQR/PQR genetic ablation, which impairs its ability to sense oxygen upshifts [9].

**Figure 3:**
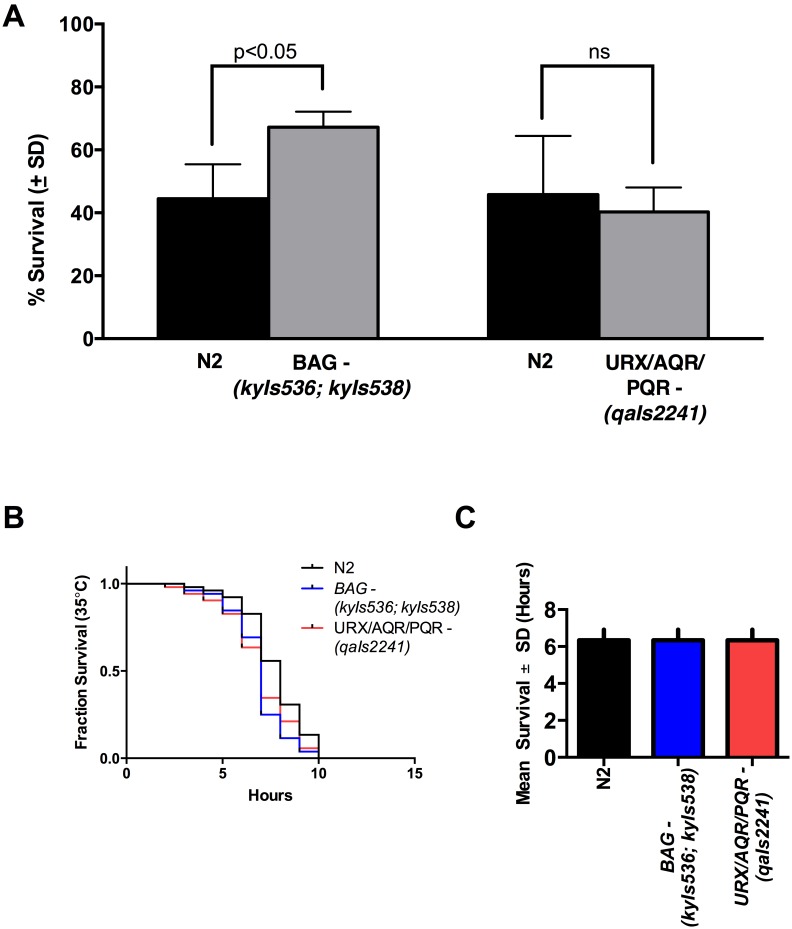
Impaired Sensing of Oxygen Downshifts Confers Survival Benefit that is Specific to Anoxic Stress. This figure shows survival results after 40–48 hours of anoxia in worms strains mutated in their ability to sense oxygen shifts. Co-bagged wild type animals (N2) were included as a control to compare mutant strain survival. The graphs depict mean survival in each strain labeled on the x-axis (light gray bars) compared to paired wild type (N2) survival (black bars). In worms with genetic manipulation that eliminates the BAG neuron, which senses oxygen shifts below the physiologic range (BAG −), there was a significant survival advantage compared to controls (67.2% vs 44.5%, *p* = 0.02). This was not seen in URX/AQR/PQR (−), which were genetically manipulated in their ability to sense oxygen shifts above the physiologic range (40.3% vs 45.8%, *p* = 0.46) (A). Lifespan (B) and mean survival (C) after heat shock lifespan was not extended in BAG (−) animals compared to N2 controls and URX/AQR/PQR (−) (*F*_(2,6)_ = 1.000; *p = *0.42). Error bars represent standard deviation. Comparative anoxia survival data were analyzed using a two-tailed paired t-test. The mean lifespan was analyzed using one-way ANOVA. The number of animals evaluated and number of trials conducted is listed in **Table 1**.

As shown in **Figure 3a**, survival after anoxia was improved in *kyIs536* (*kyIs536* vs N2, 67% vs 44%, *p*<0.05) but not in *qaIs2241* (*qaIs2241* vs N2, 40% vs 46%, *p* = 0.37).

### Survival Benefit Conferred by Impaired Oxygen Sensing is Specific

We wanted to evaluate whether the survival benefit conferred by impaired oxygen sensing was specific to anoxic stress or more generalized. To do this, we performed a heat shock survival assay. The results of this experiment appear in **Figures 3b and c** **.** We found no difference in the mean lifespan of the strain with BAG neuron ablation (*kyIs536*) compared to N2 or those worms with URX/AQR/PQR neuron ablation (*qaIs2241*). All strains had a mean lifespan of 6.3 hours (*F*_(2,6)_ = 1.000, *p* = 0.42).

### Survival Advantage with Oxygen Sensing Impairment is Dissociated from Soluble Guanylate Cyclases

Previous investigators have demonstrated that the signal transduction of oxygen shifts by oxygen-sensing neurons in *C. elegans* is accomplished through the action of specific GCs that reside in either BAG or URX/AQR/PQR [7], [9]. The GCs are heterodimeric enzymes that consist of 2 subunits and catalyze the conversion of GTP to cyclic GMP (cGMP). cGMP that is generated through the activity of these GCs then leads to depolarization of cGMP-gated ion channels that allow calcium entry and release of secondary messengers [9]. The response characteristics of the neurons are defined by their specific subpopulations of GC subunits [9]. **Table 2** lists the GCs that are resident in each of these sensory neurons. Of note, one GC, GCY-33, is common to all oxygen sensing neurons.

**Figure 4:**
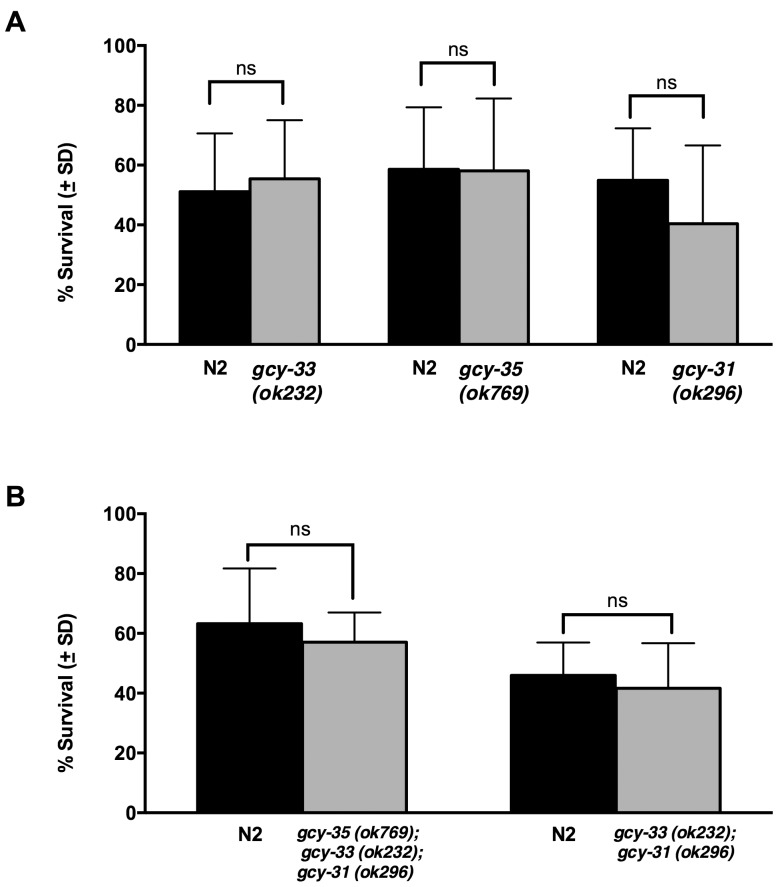
Anoxic Survival is Not Dependent on Guanylate Cyclase Activity in Oxygen Sensing Neurons. Mutant strains with null alleles in guanylate cyclases (GC) responsible for transducing oxygen shifts into neural signals (*gcy-31, gcy-35, gcy-33*) did not show significant survival differences compared to wild type (*gcy-31* vs N2, *p* = 0.15; *gcy-35* vs N2, *p* = 0.96; *gcy-33* vs N2, *p* = 0.57), suggesting that the observed survival benefit in the BAG (−) animals was not dependent on GC activity (A). Mutants with null alleles in multiple GCs also did not show significant survival benefit (*gcy-35; gcy-33; gcy-31* vs N2, *p* = 0.52; *gcy-33; gcy-31* vs N2, *p* = 0.47), demonstrating that compensated activity does not explain lack of effect seen in single mutants (B). Error bars represent standard deviation. All data were analyzed using a two-tailed paired t-test. The number of animals evaluated and number of trials conducted is listed in **Table 1**.

To evaluate the contribution of signaling that arises through this well-described system, we quantified survival after anoxia in strains of worms with null alleles in GCs specific to: 1) BAG (*gcy31*); 2) URX/AQR/PQR (*gcy35*); or 3) both BAG and URX/AQR/PQR (*gcy-33*). Survival was then compared to co-bagged N2. Results from these experiments are presented in **Figure 4a**. We found no difference in survival among these strains compared to N2 (*gcy-31* vs N2, *p* = 0.15; *gcy-35* vs N2, *p* = 0.96; *gcy-33* vs N2, *p* = 0.57). Finally, in order to evaluate the possibility of compensation from GCs that remain after single GC mutation, we used strains with null alleles in multiple GCs. *gcy-35(ok769); gcy-33(ok232); gcy-31(ok296)* contains mutations in GCs that are resident to both BAG and URX/AQR/PQR neurons. Similarly, *gcy-33(ok232); gcy-31(ok296)* contains null alleles in the two GCs found in BAG. Again, as shown in **Figure 4b**, we saw no significant difference in survival of these strains after anoxia when compared to N2 (*gcy-35;gcy-33;gcy-31* vs N2, *p* = 0.52; *gcy-33;gcy-31* vs N2, *p* = 0.47). This confluence of data leads us to conclude that the survival benefit seen in strains without BAG function is dissociated from *gcy-31, gcy-33,* and *gcy-35.*

### Impaired Neuropeptide Processing and Secretion Improves Survival after Anoxia

**Figure 5:**
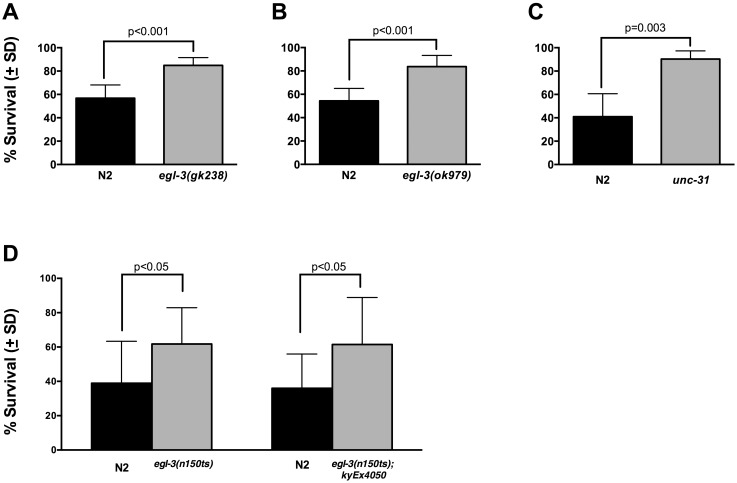
Impaired Neuropeptide Processing and Secretion Improves Survival after Anoxia and Susceptibliity is not Restored with BAG-Specific Neuropeptide Processing Rescue. Survival after anoxia in worm strains with *egl-3* null alleles (A & B), which impair neuropeptide processing. Black bars depict survival rates of co-bagged wild type (N2) animals and are compared to survival rates in mutant strains (light gray bars) labeled on x-axis. Survival is signficantly improved in *egl-*3 animals after anoxia (*egl-3(gk238)* vs N2, 84.9% vs 56.8%, *p* = 0.0003; *egl-3(ok979)* vs N2, 83.7% vs 54.4%, *p* = 0.0004), suggesting a role for neuropeptide signaling in creating anoxic vulnerability. Furthermore, inability to secrete neuropeptides via mutation of the docking protein UNC-31 also demonstrated significant survival advantage (*unc-31* vs N2, 90.3% vs 41%, *p* = 0.0034) (C). In a third *egl-3* null background, *egl-3(n150)*, there is also a survival benefit and when rescue under a BAG-specific promoter, *flp-17,* is performed via an extra-chromosomal array, there is no restoration of anoxic sensitivity (*egl-3(n150)* vs N2, 61.7% vs 38.9%, p = 0.03; *egl-3(n150); kyEx4050* vs N2, 61.5% vs 36%, *p* = 0.02) (D). Note that *egl-3(n150)* is a temperature-sensitive allele and the experiments depicted in D were conducted at 25 degrees Celsius for 24 hours. Error bars represent standard deviation. All data were analyzed using a two-tailed paired t-test. The numbers of animals trials conducted are listed in **Table 1**.

Prior work indicates that URX/AQR/PQR and BAG neurons can signal to other cells throughout the organism by the processing and secretion of neuropeptides [15], [16]. They are initially translated in pro-protein forms that are then enzymatically cleaved in several steps to their final active forms and packaged in dense core vesicles [17], [18]. Compared to traditional small neurotransmitters, they are larger and have a longer half-life [17]. Neuropeptide signaling from URX/AQR/PQR is important for behavioral response to low oxygen [16]. In order to determine if neuropeptide signaling contributes to survival after anoxia, we used two strains of worms with distinct null alleles in *egl-3*, *egl-3*(*gk238)* and *egl-3(ok979*), which encodes the principal enzyme involved in the processing of neuropeptides to their final form [19], [20]. The survival data from these experiments are presented in **Figures 5a and 5b**. We found that there was a significant survival advantage conferred after anoxia in each of these two different null alleles of *egl-3* (*egl-3(gk238)* vs N2, 84.9% vs 56.8%, *p* = 0.0003; *egl-3(ok979)* vs N2, 83.7% vs 54.4%, *p* = 0.0004). The benefit was approximately the same in each allele. We also utilized a worm strain with deletion of *unc-31(e928),* which encodes a protein that has been shown to be required for dense core vesicular fusion [21]. Survival after anoxia in these animals was also significantly improved compared to wild type (*unc-31* vs N2, 90.3% vs 41%, *p* = 0.0034) (**Figure 5c** **).** These results suggest that neuropeptide signaling contributes to anoxic response in *C. elegans*.

### BAG Specific Rescue Does not Restore Anoxic Sensitivity

Given the surivival benefit we observed from both BAG ablation as well as neuropeptide processing and secretion, we hypothesized that a neuropeptide originating in BAG neurons was the source of sensitivity to anoxia. To explore this possibility, we utilized two worm strains: 1) *egl-3(n150ts)*, a temperature sensitive null allele; and 2) *egl-3(n150ts);ky4050*, a worm strain containing an extra-chromosomal array that expresses *egl-3* under the control of the *flp-17* promoter. This will restore *egl-3* expression to the BAG neuron and the M5 pharyngeal motoneuron in this otherwise *egl-3* null background. The null allele *egl-3(n150ts)* has been shown to have limited neuropeptide expression in proteomic analysis at 20 degrees Celsius [20]. However, because this allele functions as a hypomorph at 20 degrees Celsius (M. Zimmer, personal communication and http://www.wormbase.org/species/c_elegans/strain/ MT150?query = MT150#02–10, accessed 13 May 2014), we conducted these survival experiments at 25 degress Celsius. Results of these experiments are presented in **Figure 5d**. In the *egl-3(n150ts)* background, we again saw a significant survival benefit after anoxia. Utilizing *egl-3(n150ts); kyEx4050*, which restores *egl-3* function in a BAG specific manner in an *egl-3* deficient background, we again assessed survival after 24 hours of anoxia at 25 degress Celsius. Despite restoration of neuropeptide processing activity to the BAG neuron, there was persistent survival benefit in the *egl-3(n150ts);kyEx4050* strain compared to co-bagged N2s (61.5% vs 36%, *p* = 0.02). This was comparable to the survival benefit seen when *egl-3(n150ts)* was separately compared to N2 survival after anoxia (62.7% vs 38.9%, *p* = 0.03). This suggests that protection from anoxia conferred by loss of BAG is unlikely due to the loss of a neuropeptide processed by EGL-3 within BAG neurons.

### *Daf-2* Signaling Regulates Resistance to Anoxia

**Figure 6:**
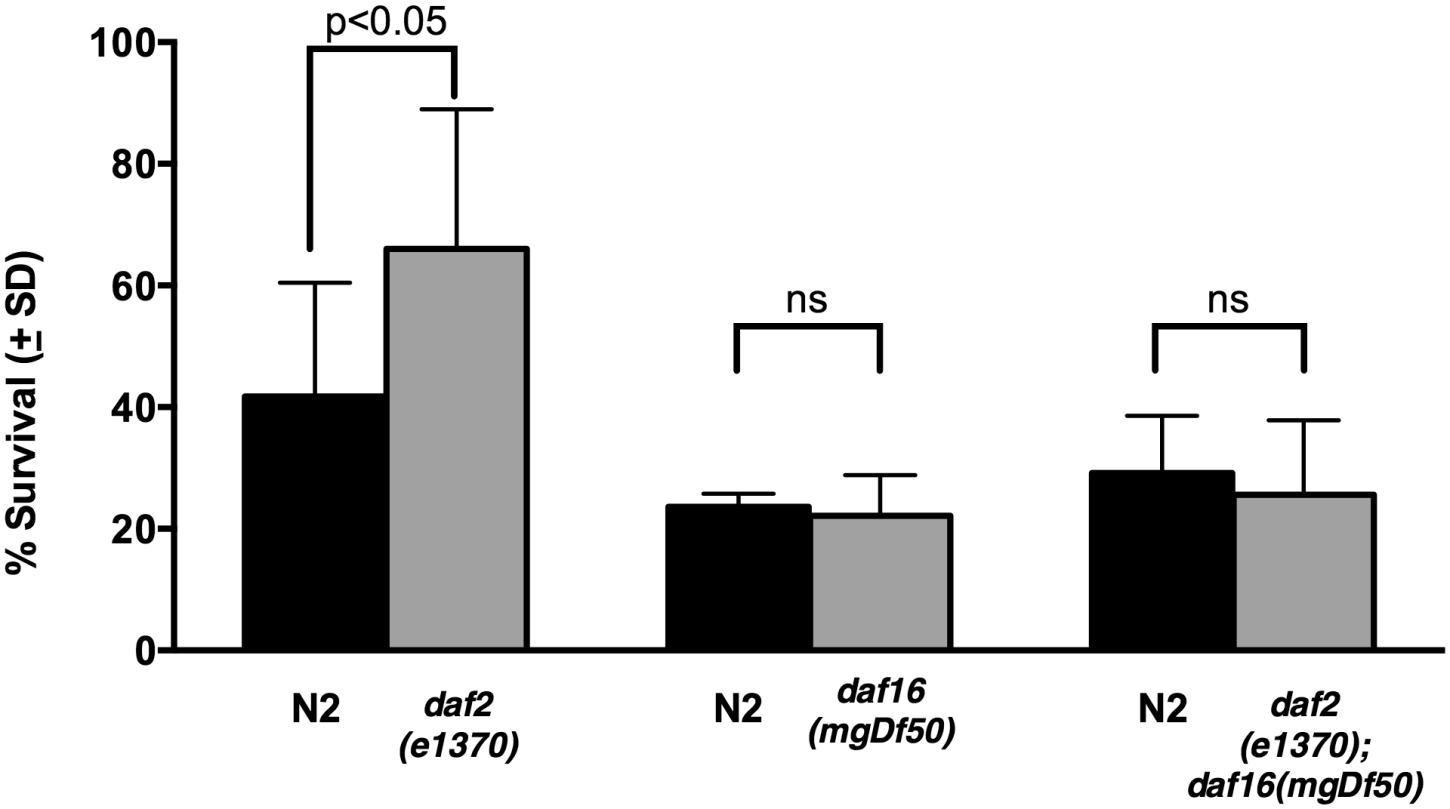
Canonical DAF-2/DAF-16 Stress Pathway Also Confers Anoxic Resistance. Survival after anoxia in worms with null alleles in the DAF-2/DAF-16 insulin signaling pathway. *daf-2(e1370)* demonstrates significant survival advantage after anoxia compared to N2 (64.6% vs 38.9%, *p* = 0.0034), which is eliminated in *daf-16(mgDf50)* (*p* = 0.73) and *daf-2 (e1370); daf-16(mgDf50)* doubles (*p* = 0.64). Error bars represent standard deviation. All data were analyzed using a two-tailed paired t-test. The numbers of animals evaluated and trials conducted are listed in **Table 1**.

Finally, we wanted to explore the contribution that the canonical stress response pathway, *daf-2/daf-16,* plays in anoxia.Since neuropeptides and insulin-like peptides are processed by *egl-3* and co-packaged into dense core vesicles, we wondered if insulin-like pepdites might be involved. The single worm IGF receptor is DAF-2. Other investigators have noted increased resistance to anoxic death in a similar system in a *daf-2* null background that is *daf-16* dependent over a range of anoxic exposures at the L2 and adult stages of development [13]. We subjected *daf-2(e1370)*, *daf-16(mgDf50),* and *daf-2(e1370); daf-16(mgDf50)* animals to anoxia for 48 hours at 20 degrees Celsius. Results of these experiments appear in **Figure 6**. There was a sigificant survival advantage in the *daf-2(e1370)* strain (*daf-2(e1370)* vs N2, 64.6% vs 38.9%, p = 0.003) that was supressed in the *daf-16(mgDf50)* null background (*daf-16(mgDf50)* vs N2, 22.1% vs 23.6%, *p* = 0.73). This confirms previous investigators’ findings in our experimental system and raises the possibility that the resistance to anoxia phenotype seen in *egl-3* mutants is due to impaired processing of insulin-like peptides in non-BAG neurons.

## Discussion

The relationship between neural oxygen sensing and organism level hypoxic injury has not been previously studied. We capitalized on the emerging understanding of oxygen sensing in the model organism *C. elegans* to begin to address this gap in knowledge. Our results suggest that oxygen sensing neurons can influence survival after anoxia in developing *C elegans.* We initially postulated that BAG neurons elaborate neuropeptide(s) that enhance the detrimental effects of anoxia on somatic tissues. However, the BAG-specific rescue of neuropeptide processing in a neuropeptide processing deficient background, *egl-3(n150ts);kyEx4050*, did not restore sensitivity to anoxia. One possibility is that these two stress responses operate in parallel to regulate susceptibility to anoxia. Alternatively, BAG neurons may signal to other cells via an EGL-3 independent manner and those cells receiving this signal may elaborate neuropeptides to regulate susceptibility to anoxia. Future experiments will be required to distinguish between these two possibilities.

Survival after anoxia varies from one experiment to the next, even within the same genotypic background. This is consistent with what other investigators using this system have described [22]. The source of this variability has eluded clear definition. One possibility is oxygen buffering by growth agar for worms. We did not measure oxygen content of the agar and this may vary from one plate to the next. Additionally, our method of inducing anoxia relied upon individual gas generators that were unique to each sealed bag and used only once. The rate at which anoxia developed from one bag to the next may not have been consistent and, while we ensure that oxygen remained below the threshold for color change of resazurin, we did not measure the exact oxygen level in each experiment. Even these small differences in oxygen may have physiologic impact in *C. elegans*, which can survive with minimal amounts of oxygen. While this variability limits comparison of absolute survival from one experiment to the next, we were able to control for it by including NGM plates with N2 worms within each bag and comparing all results to co-bagged N2 survival using paired t-tests.

Our survival data demonstrate that worms lacking the ability to sense oxygen downshift (BAG elimination) have a consistent survival advantage when exposed to anoxia. This was not seen in any other strain of worm with impairment in oxygen sensing induced by GC mutation nor by elimination of neurons sensing oxygen upshifts (AQR, PQR, URX). We anticipated that elimination of the BAG-specific GC, *gcy-31*, would phenocopy worms lacking BAG, but this was not observed. While previously published reports would suggest that neuronal function is dependent on resident GCs [9], the present observation suggests that other oxygen sensing molecules within BAG neurons may underlie this response.

The anoxic survival advantage conferred by BAG neuron elimination was unexpected, but consistent with recently published work [23]. These investigators identified URX and BAG neurons as forming a network with opposing effects on lifespan: ablation of BAG neurons increased lifespan while ablation of URX neurons decreased lifespan. Interestingly, these effects on longevity were independent of previously described stress resistance pathways, including *daf-2/daf-16*. Our finding that loss of BAG function improves survival after anoxia reinforces the beneficial role of this single neuron in combating particular stressors. This is reinforced by our finding that loss of BAG did not protect animals from heat shock; survival was the same in N2 controls and those with URX/PQR/AQR neuronal ablation. However, we did not find worsened survival after elimination of oxygen-upshift sensing neurons (URX/AQR/PQR). This may result from the method of neuronal elimination: we used strains with caspase expression in URX/AQR/PQR, thereby eliminating all of these neurons. Liu and Cai [23] used worm strains with laser ablated URX only.

There is emerging evidence that non cell-autonomous factors arising from neural networks influence stress response on a whole organism level [11], [24]. Mutations in the paired thermosensory neurons (AFD) led to reduced accumulation of *hsp70* mRNA after heat shock in multiple somatic tissues (pharynx, intestine and spermatheca) [24]. In a separate study, it was also shown that mutations in AFD neurons led to decreased protein accumulation in intestine and muscle cells in a model of protein aggregation/proteotoxicity. Furthermore, impairments in neuropeptide secretion improved this phenotype. These observations raise the possibility that signaling arising from the AFD neuron decreases organism-wide cellular reponse to proteotoxicity via a secreted neuropeptide [11]. Taken together, these results indicate that cellular responses to environmental stress can be coordinated on a somatic level by neural networks and secreted neuropeptides. Our work suggests that a similar modifying effect of neuropeptides is seen in organismal response to anoxic stress.

Recent work has demonstrated that EGL-3 plays a role in processing insulin-like peptides that serve as agonists for the insulin signaling pathway [25], [26]. Initial work published by Hung and colleagues [26] demonstrated that impaired EGL-3 function rescued aberrant synapse morphology through regulation of the insulin signaling pathway. A follow-up study [25] also demonstrated that EGL-3 is involved in processing insulin like peptides (ILP) that can serve as both agonists and antagonists of the *daf-2/daf-16* pathway. During times of stress, there is a negative regulation of agonist expression which allows antagonists to predominate and increase DAF-16 expression, which induces dauer formation. These studies taken together show that there is a role of EGL-3 function in regulating stress response through insulin signaling. Our data, as well as other invesigators’ data [13], also demonstrate a role for insulin signaling in creating resistance to anoxia. Therefore, it is possible that the survival advantage that we report in worms defective in neuropeptide processing and secretion, is in fact due to the impact of EGL-3 on insulin signaling. However, our experiments were not designed to explore this possibility. Future experiments will explore this question by evaluating the survival after anoxia in worm strains impaired in both *egl-3* as well as *daf-16*. If the survival advantage from EGL-3 impariment is solely DAF-16 mediated, we expect to rescue anoxic sensitivity. If the survival advantage persists, it suggests a role for EGL-3 processed peptides in creating susceptibility to anoxia that is independent of the DAF-2/DAF-16 pathway.

Based on these results, we conclude that oxygen sensing is involved in determining survival after anoxia, as is small peptide signaling. We hypothesize that neuropeptide signals are responsible for influencing survival after anoxia. However, our experiments were not designed to directly test this hypothesis. If these results are substantiated with future work, this may offer novel insight into oxygen sensing and anoxic survival and new opportunities for neuroprotection. Our future work will be focused on clarifying whether the effects that we attribute to neuropeptides are in fact a result of suppressed signaling through the insulin receptor or due to a distinct neuropeptide signaling pathway. Additionally, we will focus on defining the mechanism by which BAG neurons confer susceptibility to anoxia.

## Supporting Information

Figure S1 Some survival experiments were conducted with strains of worms containing the integrated array *oyIs18* for experiments that are not reported in this paper. In order to guarantee that the integrated array did not influence survival in addition to other existing genetic manipulations, we tested the survival of N2 versus *oyIs18* alone. As shown in this figure, there was no difference (*oyIs18* vs N2, 50.7% vs 42.9%, *p* = 0.35).
(TIFF)
