# Supplementary figures and images for: Oxygen Sensing Neurons and Neuropeptides Regulate Survival after Anoxia in Developing C. elegans
Source: PLoS One. 2014 Jun 26;9(6):e101102. doi: 10.1371/journal.pone.0101102 (PMC4072718; doi:10.1371/journal.pone.0101102)

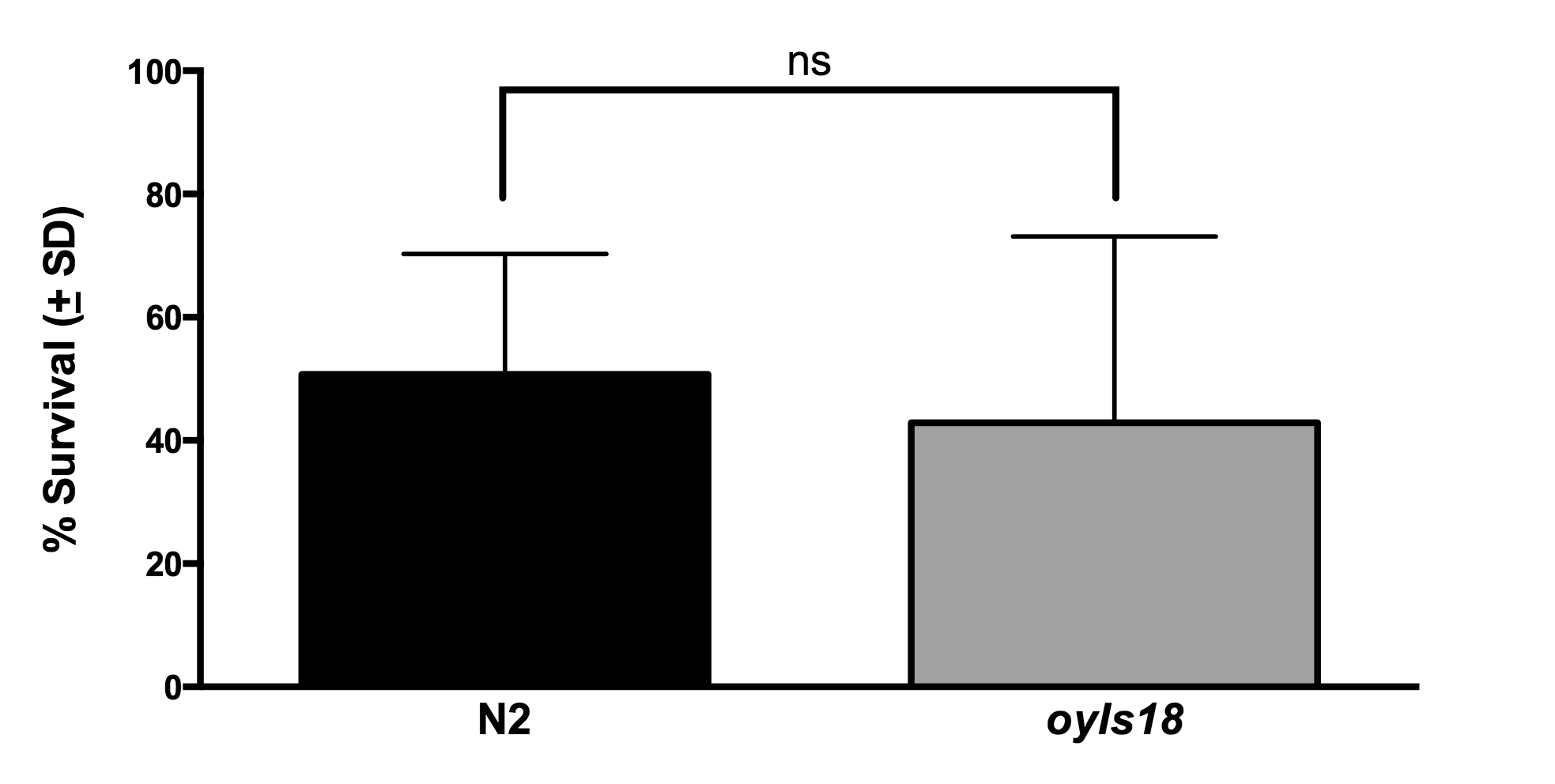

Supplement: Figure S1 — Some survival experiments were conducted with strains of worms containing the integrated array oyIs18 for experiments that are not reported in this paper. In order to guarantee that the integrated array did not influence survival in addition to other existing genetic manipulations, we tested the survival of N2 versus oyIs18 alone. As shown in this figure, there was no difference (oyIs18 vs N2, 50.7% vs 42.9%, p = 0.35). (TIFF) [file pone.0101102.s001.tiff]
